# Passive dust collectors for assessing airborne microbial material

**DOI:** 10.1186/s40168-015-0112-7

**Published:** 2015-10-05

**Authors:** Rachel I. Adams, Yilin Tian, John W. Taylor, Thomas D. Bruns, Anne Hyvärinen, Martin Täubel

**Affiliations:** Plant & Microbial Biology, University of California Berkeley, Berkeley, 94720 CA USA; Department of Civil & Environmental Engineering, University of California Berkeley, Berkeley, 94720 CA USA; Department of Health Protection, National Institute for Health and Welfare, FIN-70701 Kuopio, Finland

**Keywords:** Built environment, Dust microbiota, Electrostatic dustfall collector, Indoor microbiome, Quantitative PCR, Settled dust

## Abstract

**Background:**

Settled airborne dust is used as a surrogate for airborne exposure in studies that explore indoor microbes. In order to determine whether detecting differences in dust environments would depend on the sampler type, we compared different passive, settled dust sampling approaches with respect to displaying qualitative and quantitative aspects of the bacterial and fungal indoor microbiota.

**Results:**

Settled dust sampling approaches—utilizing plastic petri dishes, TefTex material, and electrostatic dustfall collectors (EDCs)—were evaluated in indoor spaces in the USA and Finland and in an experimental chamber study. The microbial content was analyzed with quantitative PCR (qPCR) to quantify total bacterial and fungal biomass and through high-throughput sequencing to examine bacterial community composition. Bacterial composition and diversity were similar within a sampling environment regardless of the sampler type. The sampling environment was the single largest predictor of microbial community composition within a study, while sampler type was found to have much less predictive power. Quantitative analyses in indoor spaces indicated highest yields using a petri dish approach, followed by sampling with EDCs and TefTex. The highest correlations between duplicate samples were observed for EDC and petri dish approaches, indicating greater experimental repeatability for these sampler types. For the EDC samples, it became apparent that, due to the fibrous nature of the material, a rigorous extraction protocol is crucial to obtain optimal yields and stable, repeatable results.

**Conclusions:**

Correlations between sampler types were strong both in compositional and quantitative terms, and thus, the particular choice of passive settled dust sampler is not likely to strongly alter the overall conclusion of a study that aims to characterize dust across different environments. Microbial cell abundances determined from settled dust varied with the use of different sampling approaches, and thus, consistency in the method is necessary to allow for absolute comparisons within and among studies. Considering practical aspects, petri dishes were found to be an inexpensive, simple, and feasible approach that showed the highest quantitative determinations under typical building conditions, though the choice of sampler will ultimately depend on study logistics and characteristics such as low- or high-exposure settings.

**Electronic supplementary material:**

The online version of this article (doi:10.1186/s40168-015-0112-7) contains supplementary material, which is available to authorized users.

## Background

Indoor dust is the most commonly used material to assess microbial exposures in the built environment for studies that link to human health and disease. While the relationship between actual inhalation exposure and microbial measurements from aerosols is more straightforward than for house dust, bioaerosols are highly dynamic in nature and consequently difficult to collect in a way that represents average conditions [1]. House dust is thought to be a long-term integrated sample of particles that have been airborne [2], thereby proving a composite view of microbes in the indoor environment. Another reason for the popularity of dust samples is the convenience of collection, which typically does not require costly sampling equipment and can be done in a standardized manner even by building occupants themselves and thus enables high replication, all major virtues in large epidemiological studies [3].

There are different types of house dust samples and many ways to collect a sample. Here, we differentiate between dust reservoirs, such as floors and mattresses, and airborne particles that become settled dust. Reservoirs of dust are a popular choice for collecting an integrated sample of what building occupants may be exposed [4]. However, some studies that relate different house dust sample types with bioaerosols sampled through active collection find that sampling reservoirs of dust may not closely represent airborne, inhalation exposure [5–7]. Reservoir house dust and airborne particulate matter can be disconnected for several reasons. First, there are biases in the settling of small particles, and settled communities are expected to inefficiently contain small-bodied microbes leading to their underrepresentation relative to larger bodied taxa [8, 9]. Second, in the case of floor or mattress samples, the dust also contains material tracked indoors on shoes, paws, or clothes, and in the case of mattress dust, the occupant is the major source of microbial material. Third, the time window sampled by dust reservoirs is variable and typically not precisely known.

Instead, studies assessing different indoor sampling approaches attest that a much closer representativeness of actual airborne exposure is dust that settles on a standard sampler surface located above floor level [5–7]. Passive collection on an elevated surface has two specific advantages: first, particle collection onto the standardized sampler surface occurs over a discrete and known time period. Second, placing passive samplers on a sufficiently elevated surface likely captures airborne dust rather than tracked-in, floor-based particles that may never get sufficiently airborne to contribute to human inhalation exposure. Due to these features of elevated surface samples compared to dust reservoirs, passive collectors of settled dust have been used in several studies, health-based and otherwise, to assess the microbes that occupants encounter in the built environment [10–16].

Across studies, different passive samplers have been used—samplers that vary in the nature of the material, size, and subsequent laboratory handling—and it has been questioned whether the specific sampler chosen could influence comparisons of different environments. In this study, we compare the microbial composition and quantity of settled dust that emerged when using different types of passive sampling approaches.

## Results

### Passive samplers in “real life” and experimental approaches

We employed both observational and experimental approaches to compare bacterial and fungal quantity as well as bacterial composition across sampler types. To compare the passive samplers in situ, multiple materials were used side by side in occupied buildings for 1 month across two geographic locations, the United States and Finland (Table 1). In addition, we situated different sampler types in an experimental chamber in which known and homogenous dust, collected from the vacuum bags of local homes, was aerosolized (Additional file 1). Within these different approaches, in total, five different materials were considered as passive samplers. The most basic was an empty (growth-medium-free) polystyrene petri dish [11, 12, 17], the use of which was inspired by the “pizza box” dustfall collector developed by Würtz et al. [7]. The second was a polytetrafluoroethylene fiber sampling cloth, known as TefTex, used as a surface wipe [18] in the Canadian Healthy Infant Longitudinal Development (CHILD) Study (http://www.canadianchildstudy.ca). The remaining three materials were different brands of dry sweeping cloths typically used in household cleaning: Lysol and Swiffer for the USA-based sampling and Zeeman for the Finnish-based sampling, referred to as EDC1, EDC2, and EDC3, respectively. The use of dry sweeping cloths as so-called “electrostatic dustfall collectors” (EDCs) was first reported by Noss et al. [6] and subsequently applied to study a variety of (micro)organisms and their products in settled dust [5, 10, 13, 14, 19].

**Table 1:** Summary of the different observational and experimental settings in which different passive samplers were compared

	Notation	Occupancy/input	Collection notes	Height above ground (m)	Samplers employed
USA homes	House 1	Single	4 weeks	0.3, 1.4^a^	PD(2), T(2), EDC1(2), EDC2(2)
	House 2	Couple	4 weeks	1, 2.6	PD(2), T(2), EDC1(2), EDC2(2)
	House 3	Family of five plus three dogs	4 weeks	0.5, 1.5	PD(2), T(2), EDC1(2), EDC2(2)
Finland buildings	House 1	Family of three	5 weeks	2.1^a^	PD(2), T(2), EDC3(2)
	House 2	Family of four plus dog	4 weeks	1.7	PD(2), T(2), EDC3(2)
	House 3	Couple plus two dogs	4 weeks	1.2	PD(2), T(2), EDC3(2)
	House 4	Family of three plus three dogs	4 weeks	2.0	PD(2), T(2), EDC3(2)
	House 5	Weekend residence	4 weeks	2.3	PD(2), T(2), EDC3(2)
	Office 1	Single	4 weeks	2.1	PD(2), T(2), EDC3(2)
	Office 2	Triple	4 weeks	2.1	PD(2), T(2), EDC3(2)
	Labspace	Daily use	4 weeks	2.1	PD(2), T(2), EDC3(2)
Chamber	Ch1	Dust mix 1^b^	3.17 g^c^	n/a	PD, T, EDC1, EDC2
	Ch2	Dust mix 1	2.59 g	n/a	PD, T, EDC1, EDC2
	Ch3	Dust mix 2	2.66 g	n/a	PD, T, EDC1, EDC2
	Ch4	Dust mix 2	1.78 g	n/a	PD, T, EDC1, EDC2
	Ch5	Dust mix 3	2.80 g	n/a	PD, T, EDC1, EDC2
	Ch6	Dust mix 3	3.13 g	n/a	PD, T, EDC1, EDC2

EDC1,2,3 refer to different sampler materials

*PD* petri dish, *T* TefTex, *EDC* electrostatic dustfall collector

^a^In the USA homes, four samplers were placed at two heights, for a total of eight samplers per home. In the Finnish buildings, duplicates for each of three samplers were employed at a single height, for a total of six samples per building

^b^Dust from different vacuum bags was combined into three different “dust mixes”

^c^Refers to the mass of dust input into the experimental chamber

### Bacterial composition across samplers

**Fig. 1:**
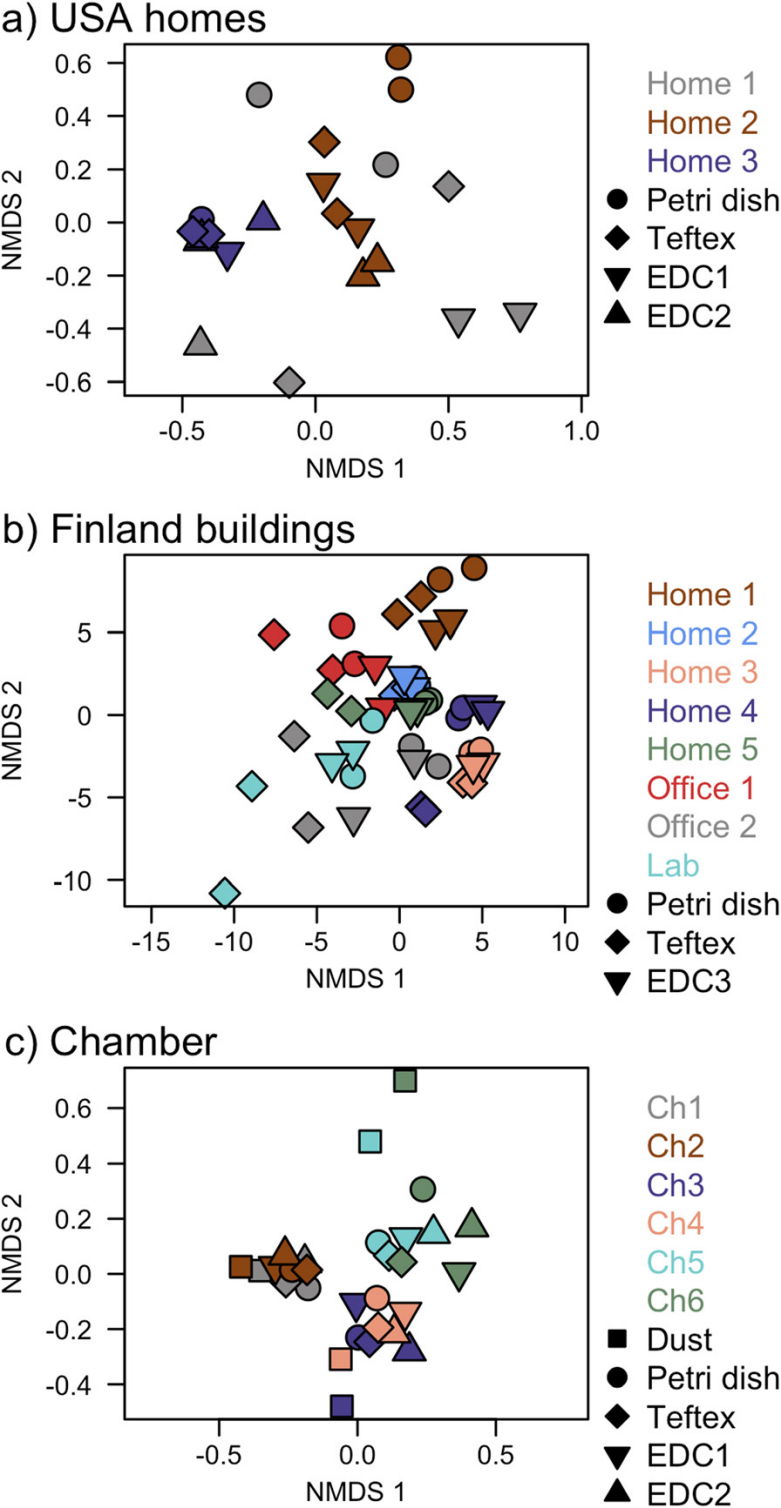
Bacterial community composition across experimental localities. Panels are **a** USA homes, **b** Finland buildings, and **c** experimental chambers, and community distances are visualized based on the Bray-Curtis community distance. Different sampling localities or rounds appear as different *colors*, and different sample types are marked with different *symbol shapes*. Except in the chamber study, samplers were tested in duplicate, so symbols will repeat

Several lines of evidence indicate that, within each experimental setting, bacterial composition was similar within a sampling environment regardless of the sampler type used to characterize that environment. That is, bacterial composition of the passively collected dust correlated most strongly with the particular environment in which the sample was collected rather than with the particular method of dust collection, and this was true both for *in situ* building samples (Fig. 1a, b) and for experimental conditions (Fig. 1c). Statistical analysis confirmed that the sampling environment was the single largest predictor of microbial community composition within a study and that sampler type was found to have much less predictive power, even if differences between sampler types reached statistical significance (Table 2). Moreover, we utilized supervised learning to determine if unlabeled communities could be classified as belonging to a particular sampler type based on a set of labeled training communities [20]. The interpretation of the technique is based on a ratio of classification error to that of baseline error. For each of the USA homes, Finland buildings, and experimental chamber, this ratio was ~1, indicating that the classifier performed no better than random guessing at which sampler types from which experimentally unlabeled microbial communities were derived (Table 2). On the other hand, the ratio of classification error to baseline error for classifying sampling environment was ≥2.3, indicating that the classifier performs at least twice as well as random guessing for determining the particular dust environment. Lastly, we examined the diversity of taxa detected in the different sampler types within a given study component (USA homes, Finland buildings, and chamber), as this study was not focused on how diversity compared across the environments. Using a mixed effect model, Shannon diversity was not found to vary across the sampler types (ANOVA *p* > 0.05), and observed richness significantly varied only in the chamber component (ANOVA *p* < 0.05), where it was lower in the EDCs compared to other sampling approaches.

**Table 2:** Factors influencing bacterial community composition in settled dust samples. Permanova analyzes the statistical variance in biological Bray-Curtis dissimilarity among bacterial communities explained by different measured variables, where *R* ^2^ represents the variance explained be each factor and the corresponding *p* value. The ratio in *supervised learning* refers to the ratio of the error in classifying microbial communities into categories of factors to the baseline error of random assignment, where a ratio of ~1 indicates no better classification than random

		Permanova		Supervised learning
Study	Factor	*R* ^2^	*p*	Ratio
USA homes	Environment	0.27	0.001	2.3
	Sampler type	0.17	0.010	0.8
	Collection height	0.04	--	0.9
Finland buildings	Environment	0.51	0.001	3.2
	Sampler type	0.06	0.001	1.1
	Replicate	0.01	--	0.6
Chamber	Round/dust mix^a^	0.43	0.001	NA^b^
	Sampler type	0.17	0.003	1.0

^a^Each mix of vacuum dust was used for two rounds of collection in the experimental chamber. Refer to Table 1

^b^Not applicable: the classification error into dust mix was 0; therefore, the ratio of baseline error to classification error was infinity

In addition, our data speak to two aspects of sampling repeatability. In the USA homes, samplers were placed at two heights, and in the Finland buildings, duplicate samplers were placed side by side at the same location. In each of these trials, duplicate samples were statistically indistinguishable with regard to bacterial composition (Table 2).

**Fig. 2:**
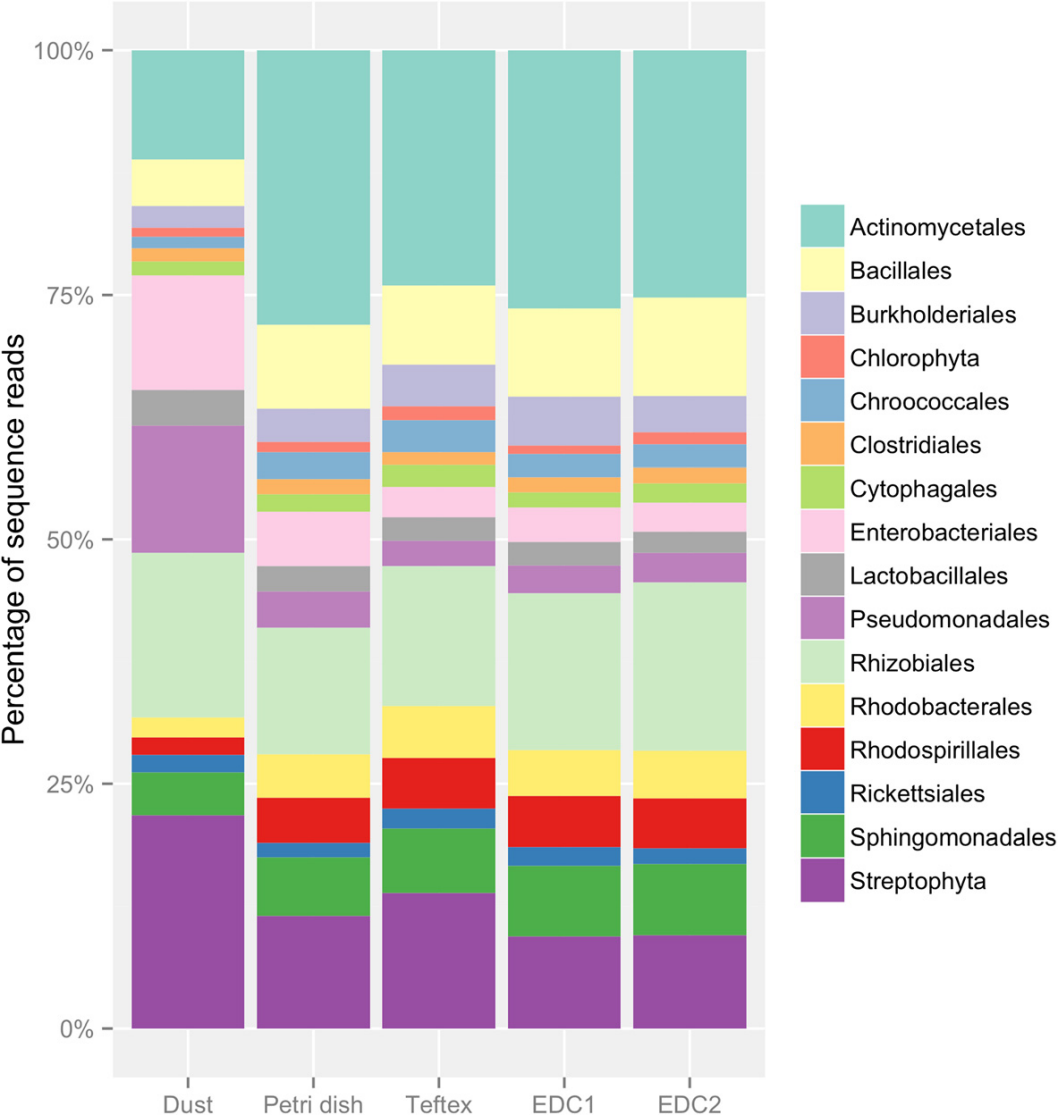
The top-most 16 bacterial orders detected in the experimental chamber. *Left column* is the input vacuumed dust, and the four *right columns* are the passively settled dust in the different sampler types

The taxonomic composition observed was largely consistent with other recent studies of indoor bacterial microbiomes (e.g., [21, 22]). Ten groups—the Staphylococcaceae, Micrococcaceae, Moraxellaceae, Corynebacteriaceae, Streptococcaceae, Sphingomonadaceae, Bartonellaceae, Enterobacteriaceae, Rhodobacteraceae, and Streptophyta—combined to ~50 % of sequence reads (Additional file 2). Within the chamber trials, for which the microbial community composition of the input dust is known through direct sequencing, there are modest differences in the compositional proportions between the vacuum dust and passive samplers. However, the passive samplers are all skewed in the same direction, such that Pseudomonadales, Enterobacteriales, and Streptophyta are underrepresented in the passive collectors, relative to their abundance in the vacuum dust that was aerosolized into the chamber (Fig. 2). Figure 2 highlights the top-most abundant taxa by sequence reads, and the full dataset is available as Additional file 2.

Within the building-based observations, taxa tended to vary in their relative abundances rather than in their detection. For example, within Finland buildings, 21 of the 25 most abundant taxa found in the petri dishes were common to the top taxa detected in the EDC and 15 were common to the top taxa in the TefTex. It was only the more rare taxa that were detected in one sampler and missed entirely in others. For instance, a bacterial operational taxonomic unit (OTU) belonging to the family Dermatophilaceae represented 0.08 % of the sequences in the Petri dish sequences and 0.004 % of the sequences in the EDC but was not detected in the TefTex samples. Within USA homes, Streptophyta (likely chloroplasts) comprised a much larger percentage of the reads in petri dishes than the other sampler types.

Fungal data were available for only one component of the study, that from USA homes. Using an approach similar to that used for bacteria, the sampling environment of the USA homes explained over half the variation in fungal composition while sampler type was not a significant predictor (see further details in Additional file 3).

### Microbial quantity across samplers

Quantitative PCR was used to estimate the microbial quantity collected in each of the samplers. Tables 3 and 4 report the bacterial and fungal counts, respectively, and additional quantitative PCR (qPCR) markers and more detailed information on analyses of the Finland building samples are included (Additional file 4). Because experimental protocols were different in the USA and Finland (see the “Methods” section), absolute values of microbial quantities across study components are difficult to compare. This was particularly the case for the extraction protocol of EDC and TefTex samplers, where the Finnish protocol included a rigorous and more efficient dust extraction procedure. In the USA homes, the highest yields of microbial biomass were found in the petri dish, followed by TefTex and the two EDCs, which had similar yields. For bacteria, the mean ratios of biomass detected relative to the highest yield in the petri dish—normalized for sampling surface area—were 0.3 for TefTex, 0.2 for EDC1, and 0.4 for EDC2; for fungi, the mean ratios were 0.2 for TefTex, 0.1 for EDC1, and 0.1 for EDC2. In the Finland buildings, the highest yields for microbial groups were generally ranked as the petri dish, EDC, and then TefTex samplers, although house 3 was an exception. For bacteria, the mean ratios of biomass detected relative to the highest yield in the petri dish were 0.4 for TefTex and 0.6 for EDC3; for fungi, the mean ratios relative to petri dishes were 0.4 for TefTex and 0.8 for EDC3. The relative differences across locations matched predictions based on occupancy, although we acknowledge low sample numbers. For example, within the USA, quantities were lowest for house 1, which was occupied by a single occupant, and highest for house 3 occupied by a family of five with three dogs. In Finland, houses showed higher microbial biomass than work settings (one labspace, two offices). In contrast to the home settings, yields from the chamber did not show such clear trends. In the chamber, which had much higher particle loading onto the samplers compared to the buildings, TefTex samplers most often showed the highest yields, followed by the petri dish samplers. For bacteria, the mean ratios of biomass detected relative the highest yield in TefTex were 0.7 for petri dish, 0.5 for EDC1, and 0.2 for EDC2; for fungi, the mean ratios were 0.7 for petri dish, 0.5 for EDC1, and 0.2 for EDC2.

**Table 3:** Bacterial quantity across sampler types and experimental conditions. Values reported are mean and standard deviations of cell equivalents per 100 cm^2^ of sampler per time of exposure (day for USA and Finland, hour for chamber). Note that bacterial determinations relied on different qPCR protocols in the USA/chamber studies and the study part in Finland, and thus, absolute values are not well comparable between study parts but are comparable between sampler types within environment

	Environment	Petri dish	TefTex	EDC1	EDC2
USA	Blank	0.2 (0)	0.1 (0)	0.1 (0)	0.1 (0.1)
	House 1 low	12 (3)	7 (4)	--^a^	14 (8)
	House 2 low	380 (110)	21 (7)	13 (5)	17 (5)
	House 3 low	690 (120)	25 (14)	38 (39)	--^a^
	House 1 high	20 (4)	8 (2)	12 (8)	8 (1)
	House 2 high	33 (12)	14 (4)	7 (1)	12 (8)
	House 3 high	270 (160)	19 (9)	14 (10)	42 (60)
		Petri dish	TefTex	EDC3	
Finland^b^	Blank	11 (6)	9 (13)	17 (24)	
	House 1	2000 (900)	310 (89)	820 (280)	
	House 2	2800 (280)	890 (280)	2100 (240)	
	House 3	1700 (170)	2200 (260)	1900 (24)	
	House 4	3100 (140)	610 (120)	2600 (440)	
	House 5	2800 (780)	610 (110)	1400 (560)	
	Office 1	540 (150)	66 (89)	180 (40)	
	Office 2	660 (590)	100 (3)	400 (55)	
	Labspace	220 (40)	78 (65)	73 (11)	
		Petri dish	TefTex	EDC1	EDC2
Chamber	Round 1	410 (41)	1200 (360)	570 (140)	320 (47)
	Round 2	670 (110)	610 (26)	190 (24)	100 (4)
	Round 3	140 (34)	260 (85)	250 (29)	87 (16)
	Round 4	610 (54)	490 (41)	200 (21)	160 (14)
	Round 5	630 (41)	1200 (520)	270 (120)	110 (7)
	Round 6	120 (6)	290 (20)	100 (12)	91 (9)

*EDC* electrostatic dustfall collector

^a^There were missing wipe samples for two of the houses

^b^Bacteria in Finland samplers are represented as Gram-positive bacteria in cell equivalents. Assays for Gram-negative bacteria are included in Additional file 4

**Table 4:** Fungal quantity across sampler types and experimental conditions. Values reported are mean and standard deviations of cell equivalents per 100 cm^2^ of sampler per time of exposure (day for USA and Finland, hour for chamber). Note that fungal determinations relied on different qPCR protocols in the USA/chamber studies and the study part in Finland, and thus, absolute values are not well comparable between study parts but are comparable between sampler types within localities

	Environment	Petri dish	TefTex	EDC1	EDC2
USA	Blank	1 (0.3)	0.7 (0)	ND^a^	ND
	House 1 low	7 (5)	0.8 (0.3)	--^b^	0.3 (0.2)
	House 2 low	120 (11)	37 (15)	10 (2)	33 (14)
	House 3 low	490 (100)	28 (5)	14 (0.5)	--^b^
	House 1 high	14 (5)	3 (3)	0.9 (1)	0.6 (0.1)
	House 2 high	130 (20)	27 (14)	11 (1)	10 (2)
	House 3 high	260 (93)	25 (1)	14 (0.5)	3 (1)
		Petri dish	TefTex	EDC3	
Finland	Blank	ND	ND	0.9 (1)	
	House 1	66 (12)	35 (20)	48 (12)	
	House 2	100 (8)	45 (11)	76 (7)	
	House 3	240 (57)	210 (6)	380 (57)	
	House 4	210 (27)	85 (13)	190 (18)	
	House 5	350 (26)	78 (4)	210 (49)	
	Office 1	54 (19)	6 (0.3)	40 (0.6)	
	Office 2	26 (16)	12 (1)	24 (3)	
	Labspace	14 (2)	6 (3)	6 (3)	
		Petri dish	TefTex	EDC1	EDC2
Chamber	Round 1	120 (38)	1100 (720)	470 (430)	220 (130)
	Round 2	680 (150)	1300 (560)	330 (230)	57 (7)
	Round 3	31 (5)	29 (7)	51 (14)	19 (15)
	Round 4	94 (23)	130 (20)	11 (5)	15 (5)
	Round 5	240 (86)	240 (23)	31 (10)	14 (7)
	Round 6	61 (30)	61 (5)	15 (3)	15 (3)

*EDC* electrostatic dustfall collector

^a^None detected

^b^There were missing wipe samples for two of the houses

Side-by-side samplers in the Finland component of the study allows for examination of the correlation between duplicate samplers. Table 5 summarizes Pearson’s correlations of duplicate sampler qPCR determinations. Overall, strong and highly significant correlations were observed for the duplicate determinations in most cases, except in some cases for the TefTex material. The highest correlations were found for EDC3, followed by petri dish, and then TefTex. Although limited by a small number of different sampling environments and duplicate samples, analyses of the intraclass correlation (ICC, comparing the within-location variance to between-location variance) and coefficient of variation (CoV) of duplicates showed similar trends, with highest correlation/lowest variation observed for EDC3, followed by petri dish sampling, then the TefTex material. Lastly, correlations of biomass determinations *between* different sampler types were strong (Pearson correlation >0.85 for each sampler pairwise correlation). Further information is detailed in Additional file 4.

**Table 5:** Pearson correlation coefficients of naturally log-transformed qPCR data for duplicate determinations from sample pairs in Finland locations

qPCR assay	Pairs of duplicates	Petri dish	TefTex	EDC3
Total fungi	8	0.95***	0.96***	0.97***
Penicillium/Aspergillus spp.	8	0.96***	0.53	0.97***
Gram-positive bacteria	8	0.85**	0.83*	0.97***
Gram-negative bacteria	8	0.94***	0.64	0.97***
All qPCRs combined	32	0.96***	0.76***	0.97***

*p < 0.05; **p < 0.01; ***p < 0.001

## Discussion

Passive collection of dust settled over a defined period represents a valuable tool for assessing microbial exposures in indoor environments, and this study sought to examine how the choice of passive sampler could affect estimates of the community composition and microbial biomass from the settled dust of different environments. We found that, for a given dust environment, estimates of bacterial community composition and diversity in passively collected airborne dust were similar regardless of the sampler type, as were estimates from our smaller study of fungal community composition. In the experimental chamber study, we did note an underestimate of some groups of bacteria, Pseudomonadales, Enterobacteriales, and Streptophyta, relative to the vacuum dust used in the dispersion, but the underestimation was similar for all collection methods. In contrast, estimation of the quantity of microbes was more sensitive to differences in both the dust loading of the environment and the experimental procedures used to collect, extract, and process the dust from the samplers. We discuss three areas of the experimental pipeline in which the different sampler types could vary in their efficiencies: collection, retention, and extraction.

For collection efficiency, we refer to the properties of the sampler itself for collecting settling dust. For instance, the electrostatic properties of some surfaces could potentially bias the kind of settling particles that deposit. Many microbial spores carry a small net electrical charge, either positive or negative, although it is generally thought that most are slightly negative [23]. A similarly negatively charged sampler surface could repel particles. All sampler types used here are electronegative to varying degrees [18, 24], but it is unclear how much charge the samplers retain after heat treatment, if used, or after time employed in the field. Another property of the sampler that could affect collection is whether the material is likely to become saturated, thereby preventing further dust collection. It remains to be tested whether the small bias observed in the collection of some bacteria taxa in passive samplers relative to the source dust (Fig. 2) is a consequence of disproportional aerosolization of the source dust, size dependence of particle settling, surface charge of the sampler relative to the surface charge of the bioaerosols, or some other process.

Another component of sampling efficiency is related to the retention of particles once collected or whether the forces generated by air speeds indoors are sufficient to overcome the adhesion forces between particles and passive collection surfaces. There are observations that the release of dust collected on “smooth” surfaces, such as petri dishes, are greater than from fibrous materials such as TefTex and EDCs [5]. However, the microbial compositions in cow stables were similar between a plastic passive sampler and an electrostatic wipe [19]. Under experimental conditions, resuspension of particles has been studied at air speeds [25] that are orders of magnitude higher than the typical range of speeds in indoor air [26]. In a typical household, the likelihood for a passive sampler to encounter air speeds sufficient to resuspend particles likely depends on the location of the sampler with regard to occupant movements and ventilation strategies.

Lastly, the release of biological material from the sampling matrix and subsequent collection is the dominant factor affecting the extraction efficiency of dust and associated microbial material. In all samplers, the dust must first be isolated from the sampler, and in this study, the quantity of airborne dust in the experimental system affected the quantitative estimates that resulted. Within the building-based trials, under levels of particle loading typically encountered in the built environment, the petri dishes almost always yielded higher cell abundance than TefTex or EDCs (Tables 3 and 4), likely due to the simple process of using a swab to recover microbes from the sampler. The step of pre-extraction of the dust from the fabric-based samplers (TefTex and EDCs) requires specialized equipment and suspension in buffers. A more rigorous microbial recovery process that was employed in Finland, as compared to the USA (see the “Methods” section), narrowed the gap in recovery between plain petri dishes and EDCs. In the chamber system, particle loading was much higher than representative conditions. For instance, with 1.77 g of dust fed, the surface dust loading at the bottom of the chamber was approximately 2.3 g/m^2^. With a typical dust fall rate in residences of ~0.005 g/(m^2^ ∙ day) [27], it would take approximately 460 days to reach this level of dust in the sampler. Under this high particle loading such that a thick layer of dust was left in the samplers (Additional file 1), one swab was insufficient to remove all the dust from one petri dish, resulting in an underestimation of microbial biomass per petri dish.

As microbial differences across different environments were detectable with each of the passive sampling methods tested here (despite potential differences in efficiencies just discussed), another consideration is the practical implications of employing the different samplers in field studies. Each sampler had limitations in particular aspects (Table 6). For instance, sampling materials will vary in their ease of acquiring, preparing, and shipping the material. More importantly, however, are the different protocols—and accompanying equipment—required for isolating the dust from the samplers. The pre-extraction steps of the dust from the fabric-based samplers increase the time and expense of the protocol compared to the petri dish protocol. Considering the economics of implementing and processing the samplers in light of the composition and quantitative results here, petri dish samplers represent a robust method for passive dust collection, although the extraction process may require some additional labor in high particle loading environments compared to more typical building environments.

**Table 6:** Comparison of handling requirements for the different samplers tested in this study

Sampler	Acquiring material	Preparation for field-sampling and sampling	Sample pre-extraction: specificities and equipment	Concerns with regard to particle loading
Petri dish	Purchase from general laboratory supply	Must ship securely so samplers do not break in transport	Swab to collect dust material from sampler; no sample pre-extraction so direct DNA extraction possible	Appears to perform well under low and moderate particle loading. Under high particle loading, dust collection may require several collection swabs
TefTex	Purchase from manufacturer of industrial fabrics	Cutting material to desired sampler size and, if desired, place in holder; heat treatment to reduce background may be required for some biochemical determinations	Pre-extraction in buffer necessary (10+ mL); ideally sonicator for extraction	Requires no difference in treatment under different particle loads
EDCs (various materials used as wipes)	Purchase from manufacturer or from stores that sell household cleaning supplies	Cutting material to desired sampler size and, if desired, place in holder; heat treatment to reduce background may be required for some biochemical determinations	Relatively large pre-extraction buffer volumes needed (10–50 mL); ideally stomacher for rigorous extraction	Requires no difference in treatment under different particle loads

## Conclusions

Passive collection of dust settling into sampler over a month, or similar period, is a method of detecting differences in aerosolized microbial communities that accounts for temporal variation in bioaerosol concentration and composition in real-world settings. Ideally, the sampler would be inexpensive in equipment and analysis, facilitating high replication necessary for epidemiological and ecological research. Our study points to empty, plastic petri dishes at meeting these criteria. The determination of microbial community composition was little affected by the exact material nature of the passive sampler, whether the amount of microbial biomass was typical of that encountered in the built environment or higher. However, determination of microbial biomass was underestimated in petri dishes when the amount of biomass in the dish was higher than typically encountered in the built environment, an underestimate that likely could be corrected by employing two swabs instead of one to isolate the dust. While the choice of passive sampler will ultimately depend on study logistics and characteristics, our results indicate that, under typical building conditions, using petri dishes for collection of airborne settled dust is a simple approach that will reliably capture the different microbial profiles across indoor environments.

## Methods

### Sample collection

For the USA-based study, homes in the San Francisco Bay Area of California were sampled in October 2014. Samplers employed were the empty petri dish, or petri dish containing a TefTex, EDC1 (Lysol brand), or EDC2 (Swiffer brand) pad. TefTex was provided as a 39.1-cm^2^ piece, having been heat-treated at 250 °C for 2.5 h and packed aseptically. The EDC materials were cut into 42.3-cm^2^ square pieces of fabric and autoclaved at 250 °C in aluminum foil for 20 min. Images of the sampling devices employed in house 2 are shown as Additional file 5. After exposure, petri dish holders were closed.

In Finland, samplers employed were the petri dish, TefTex, and EDC3 (Zeeman). The experimental locations were five homes (all in the living room), two office rooms, and one laboratory setting, sampled along with field blanks during December 2014 and January 2015. Sampling duration was 4–5 weeks at a height between 1.2 and 2.3 m from the floor. Petri dishes were opened and applied as such, while the TefTex wipes were placed into sterile, opened glass petri dishes. EDC3s were heat-treated at 200 °C for 4 h and mounted into a plastic frame, where the exposed area was 206 cm^2^ [6]. TefTex wipes were transferred straight at the end of sampling into sterile Stomacher rollbags (Interscience), and EDC frames were closed at the end of sampling and stored closed in sterile plastic bags, before transfer into rollbags for further processing.

The experimental chamber was designed as a closed system in which to subject passive samplers to a defined and uniform aerosol source (Additional file 1). Compressed air first passed through a HEPA filter and then a glass jar containing dust. The air with the suspended dust was next passed through a neutralizer and then to a bench-top-sealed cylindrical brass chamber in which samplers were situated at the bottom. Compressed air was input until all the vacuum dust in the glass jar was entered into the system. A fan situated inside the chamber created well-mixed conditions during inoculation lasting <5 min, and then, the fan was turned off and the system left still for 5 h to allow particles to settle. Sieved household vacuum dust was used as the dust source and compositionally analyzed separately.

### DNA extraction

All samplers were stored at room temperature until processing. The USA homes and chamber-based studies were extracted in one lab, and the Finland building samples in another. In both labs, all interior surfaces of the petri dish samplers were swabbed thoroughly with a sterile cotton swab wetted in sterile water + 0.05 % Tween 20. Immediately afterward, sterile scissors were used to cut the cotton from the swab stick and place the tip into a glass-bead filled tube [11].

In the USA-based lab, TefTex and EDCs were suspended in 15 mL falcon tubes with 10 mL of sterile water + 0.05 % Tween 20 and shaken for 1 h. The sampler material was removed, and the release microbes concentrated at 2000 g for 1 min. In the case of TefTex, the full volume of buffer remained in the tube due to the hydrophobic properties of the material, while for the EDCs, some buffer was retained in the material when removed. The settled material was put into a glass-bead filled tube, and here, the extraction protocol across sampler types converged [22]. Briefly, samples were bead-beaten for 1 min with Miller buffers and then exposed to another minute of bead-beating after the addition of phenol:chloroform:isoamyl alcohol. The supernatant was then processed with the MoBio PowerSoil Kit starting with the C4 step.

Extraction from the materials in Finland followed a more rigorous extraction protocol recently described for a study in New Zealand homes [16]. For TefTex, each wipe was extracted twice in 15 mL sterile water + 0.05 % Tween 20. Extraction in the rollbags was done in a stomacher (a paddle blender homogenizer typically used in food science) for 10 min per extraction; duplicate extracts were combined into one 50-mL screw cap tube and concentrated via centrifugation (6000×g, 15 min, 4 °C) to 1000 μL. Aliquots of the TefTex extracts were stored at −20 °C until DNA extraction; 500 μL of TefTex extract was subjected to DNA extraction. EDC wipes were transferred in the laboratory from the plastic holder into sterile stomacher rollbags. Wipe extraction was performed two consecutive times per each wipe in 30 mL sterile water + 0.05 % Tween 20. Extraction in the rollbags was done in a stomacher for 10 min per extraction; duplicate extracts were concentrated via centrifugation (6000×g, 15 min, 4 °C) and combined to a final volume of approximately 1500 μL. Aliquots of the EDC extracts were stored at −20 °C until DNA extraction; 500 μL of EDC extract was subjected to DNA extraction. Extraction was performed using beat milling for mechanical cell disruption and subsequent DNA purification as described previously [28], with minor modifications. DNA cleanup was performed using Chemagic DNA plant kit with DNAeX-treated magnetic beads on KingFisher DNA extraction robot. We added salmon testis DNA to the samples prior DNA extraction as internal standard [29] to control for differences in DNA extraction efficiencies and inhibition in qPCR. We note that the use of a sonicator is recommended for extraction from the TefTex but was not used here in either extraction protocol [18].

### Sequencing and bioinformatic analysis

Following DNA extraction, all samples were processed together for compositional analysis. Primers adapted for Illumina MiSeq sequencing, as developed by the Earth Microbiome Project [30], were used to amplify bacterial DNA. One microliter of DNA (concentration not determined) was combined with 2.5 μL 10× HotStarTaq Buffer, 0.13 μL HotStarTaq, 0.5 μL of 2 μM dNTPs, 1 μL each of 10 μM forward primer and reverse primer, 0.25 μL of 100 mg/mL BSA, and 17.6 μL water to 25 μL reaction. Thermocycler protocols involved heating at 95 °C for 5 min followed by 35 cycles at 95 °C for 30 s, at 50 °C for 30 s, and at 72 °C for 1 min, ending with a final extension at 72 °C for 10 min. Samples were amplified in triplicate and pooled prior to cleanup with Ampure Beads. Quantification was determined using the Qubit and hsDNA reagents, and samplers were pooled for MiSeq sequencing (2 × 250 paired-end) at the Vincent J. Coates Genomics Sequencing Laboratory at University of California Berkeley, supported by NIH S10 Instrumentation Grants S10RR029668 and S10RR027303.

Bioinformatic analysis relied on the open-source software QIIME [31]. Using the R1 reads with default quality filtering, operational taxonomic units (OTUs) were chosen using open reference picking. Although these conditions have been shown to lead to inflated OTU counts relative to other bioinformatic approaches [32], the results and conclusions of this study are not expected to be impacted. Chimeric OTUs were identified using Chimera Slayer. OTUs identified as chimeric and those not aligning to the Greengenes database [33] at 85 % were removed. A minimum of three observations was required for an OTU to be retained. Based on the negative control samples, we removed the 23 OTUs most abundant by sequence reads in the negative controls, representing 78 % of the negative control sequences by read abundance. All the negative control samples were excluded when the resulting OTU was rarefied to 6500 sequences per sample (Additional file 2). After these quality-filtering steps, the resulting OTU table contained 929,500 sequences comprising 25,800 OTUs.

Composition analysis was implemented in R [34] and relied on the qiimer, biom, vegan, and nlme packages. Permutation analysis of variance (permanova, implemented as “adonis”) was used to partition the community distance matrices among the sources of variation. Shannon and observed richness were compared across sampler types using a mixed effect model with the sampler type as a fixed effect and the sampling location as a random effect. QIIME [31] was used for supervised learning and summarizing taxonomic assignments. Fungal analyses relied on a similar approach of clustering OTUs followed by taxonomic assignment and are explained in Additional file 3. As with bacteria, permanova was used to determine how variance in sampling environment and sampler type explained fungal community composition.

### Quantitative PCR

For the USA homes and chamber samples, qPCR followed previous protocols [17], with primers FF2/FR1 as universal fungal primers targeting the large ribosomal subunit gene and 27F/518R targeting a region of the 16S ribosomal gene. Standard curves for fungi relied on extraction of a known quantity of *Penicillium purpurogenum* spores and for bacteria on *Pseudomonas syringae*. Quantitative PCR protocols applied to samples in Finland were performed as described previously [31]. Total fungal DNA, as well as DNA of Penicillium spp./Aspergillus spp./Paecilomyces variotii group (PenAsp), and Gram-positive and Gram-negative bacteria cell abundances, were assessed [28, 35, 36]. Standard curves for the Pen/Asp group were produced using DNA extracted from five pure strains (*Penicillium brevicompactum*, *Aspergillus ochraceus*, *Penicillium chrysogenum*, *Aspergillus versicolor*, and *Aspergillus fumigatus*) and for total fungal assay, an additional two strains (*Cladosporium herbarum* and *Cladosporium cladosporioides*). For the Gram-positive/Gram-negative bacterial assays, standard curves were done from a bacterial mixed culture [28] including the Gram-positive bacterial species *Staphylococcus aureus*, *Streptomyces californicus*, and *Bacillus subtilis*, as well as the Gram-negative *Escherichia coli*, *Sphingomonas faeni*, and *Pseudomonas aeruginosa*.

### Availability of supporting data

The raw sequences supporting the results of this article are available in the NCBI’s Sequence Read Archive (SRA) repository as SRP062794.

## Additional files

Additional file 1:
**Experimental chamber for soiling passive samplers.** Graphic depiction of the operation of the chamber system. Figure S1: schematic of the dust aerosolization-deposition system. Table S1: results of spatial distribution test. Table S2: experimental log. Figure S2: passive samplers after experimentation showing the high dust loading. Additional file 2:
**Bacteria OTU table.** The file contains the full dataset of bacterial orders detected. Additional file 3:
**Fungal community composition for the study in USA homes.** Description of the experimental methods and results for high-throughput sequencing of the fungi detected in different passive samplers. Additional file 4:
**Further quantitative characterization of fungi and bacteria with qPCR in Finnish indoor spaces.** Details on the complete qPCR measurements taken. Table S1: microbial quantity (cell equivalents/100 cm^2^ material) as determined through qPCR. Table S2: qPCR geometric mean levels (min-max) of microbial determinations from settled dust utilizing different passive sampling devices in eight homes (duplicate samples). Table S3: intraclass correlations coefficients (ICC) of naturally log-transformed qPCR data for duplicate determinations from eight sample pairs. Table S4: mean coefficient of variations for individual qPCR determination for different sampler types (duplicate samplers of each type in eight homes) and for all qPCR determinations combined. Additional file 5:
**Picture of the samplers deployed in USA house 2.** Demonstration of the samplers employed in a USA home-based study.

## Abbreviations

EDC: electrostatic dustfall collector
PD: petri dish
T: TefTex
