# Supplementary material for: Passive dust collectors for assessing airborne microbial material
Source: Microbiome. 2015 Oct 5;3:46. doi: 10.1186/s40168-015-0112-7 (PMC4593205; doi:10.1186/s40168-015-0112-7)
Supplement: Additional file 1: — Experimental chamber for soiling passive samplers. Graphic depiction of the operation of the chamber system. Figure S1: schematic of the dust aerosolization-deposition system. Table S1: results of spatial distribution test. Table S2: experimental log. Figure S2: passive samplers after experimentation showing the high dust loading. [file 40168_2015_112_MOESM1_ESM.docx]

**Additional file 1: Experimental chamber for soiling passive samplers**


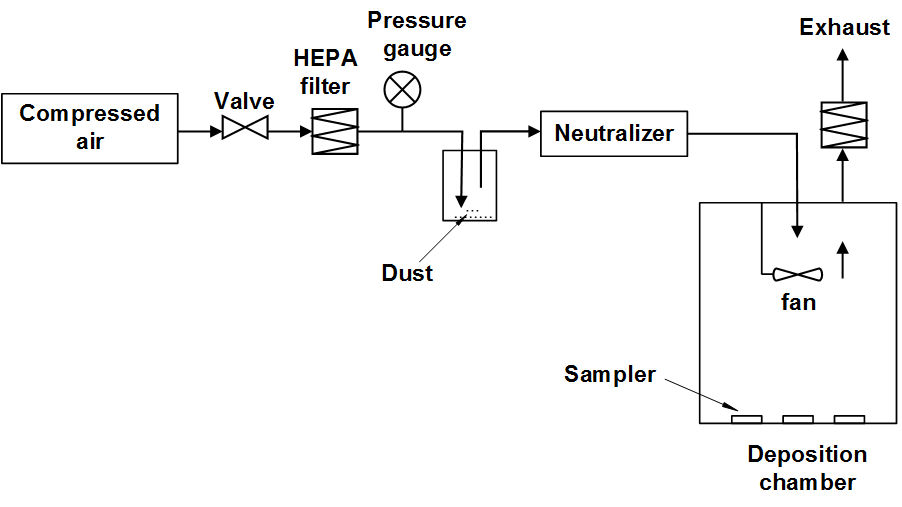
Vacuum bags were collected from eight houses around Berkeley, CA. House dust retrieved from the vacuum bags was sieved to 38 µm with a series of U.S. standard sieves and stored under room temperature. As shown in Figure S1, a dust aerosolization-deposition system was built to deposit house dust onto passive samplers. House dust placed in a sealed glass jar was aerosolized by filtered compressed air at 36 psi. The particle-laden air then passed through an Aerosol Neutralizer (Model 3770, TSI Inc., Shoreview, MN, USA) to ensure a Boltzmann charge distribution, as the particles are usually electrostatically charged during dispersion. The particles were then injected into a cylindrical deposition brass chamber (D: 25.4 cm, H: 32 cm) at the center of the ceiling, while a small fan was blowing upward to create a well-mixed condition. Passive samplers were distributed at the bottom of the deposition chamber around the rim at four different locations, and a maximum of four samplers could be soiled per time. After dust injection, the system was left still for a 5-hr period to allow particles to settle.

Figure S1. Schematic of the dust aerosolization-deposition system.

To evaluate the spatial distribution of the deposited dust, four 7 cm aluminum petri-dishes were placed at the bottom of the deposition chamber. The amount of dust collected on each petri-dish was determined by the mass difference before and after soiling. Because the amount of less than 38 µm dust is rather limited, the 38< d_p_ <106 µm fraction was also used to test the spatial distribution. As shown in Table S1, the difference in mass loading at different locations on the bottom of the chamber was less than 8%.

Table S1. Results of spatial distribution test.

| Run # | Particle size d_p_ (µm) | Dust fed (g) | Loading (mg) | Coefficient of Variation (n=4) |
| --- | --- | --- | --- | --- |
| A | 38< d_p_ <106 | 1.13 | 5±0.41 | 8% |
| B | d_p_< 38 | 1.77 | 8.86±0.41 | 5% |

For the experimental conditions, three batches of dust were used: Dust from House 1 was used for Run 1 and 2, mixed dust from many houses were used for Run 3 and 4, and a new batch of mixed dust was used for Run 5 and 6. Further details on the experimental condtions are included in Table S2.

Table S2: Experimental log

| Run # | Date | Dust (g) | Settling time (h) | Location 1 | Location 2 | Location 3 | Location 4 |
| --- | --- | --- | --- | --- | --- | --- | --- |
| 1 | 05/21/15 | 3.17 | 5-5.5 | TefTex | EDC 2 | EDC 1 | Petri dish |
| 2 | 05/27/15 | 2.59 | 5 | Petri dish | TefTex | EDC 2 | EDC 1 |
| 3 | 05/28/15 | 2.66 | 5 | EDC 1 | Petri dish | TefTex | EDC 2 |
| 4 | 05/29/15 | 1.78 | 5 | EDC 2 | EDC 1 | Petri dish | TefTex |
| 5 | 06/16/15 | 2.80 | 5 | EDC 1 | Petri dish | TefTex | EDC 2 |
| 6 | 06/17/15 | 3.13 | 5 | EDC 2 | EDC 1 | Petri dish | TefTex |


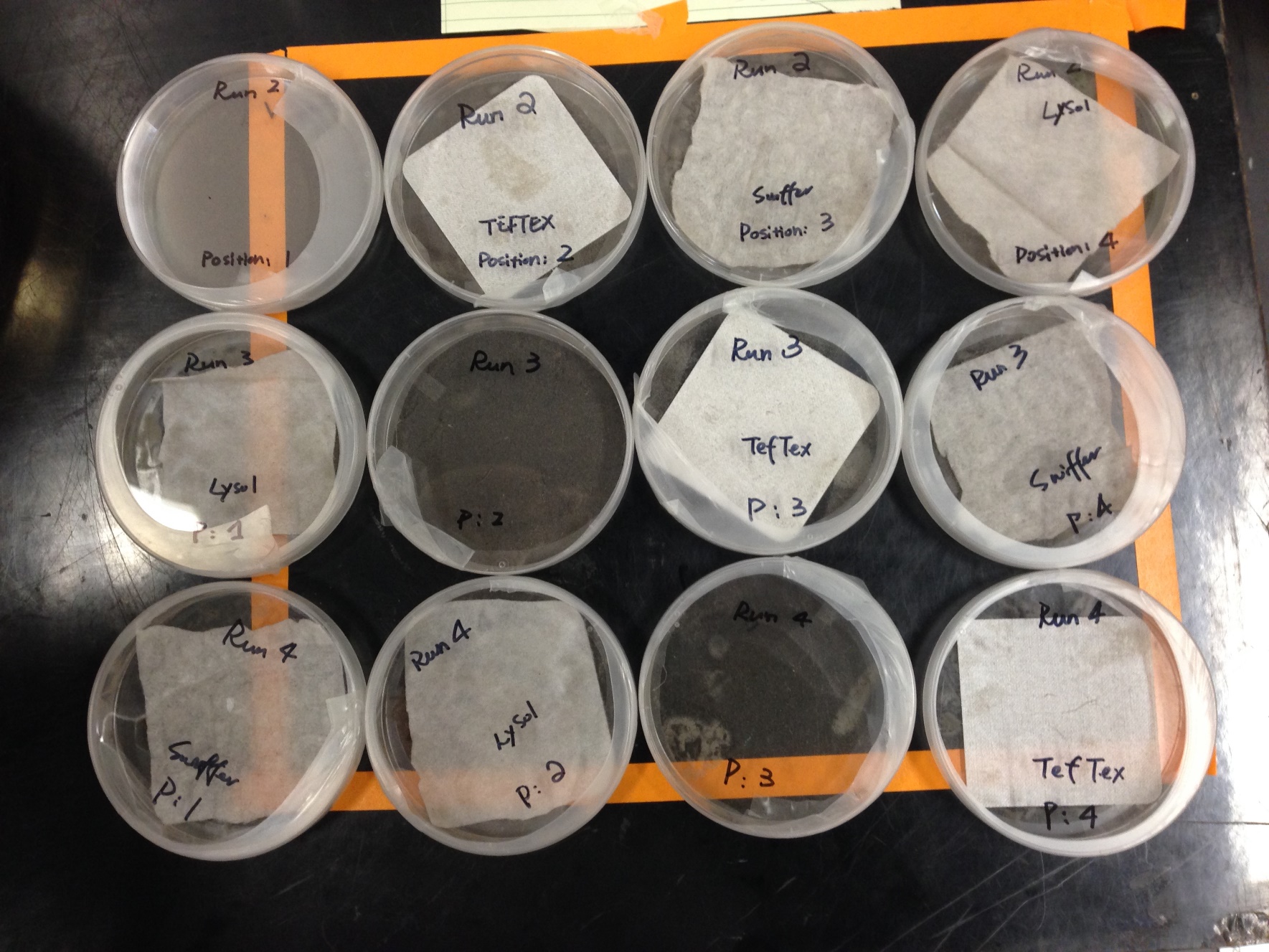


Fig S2. Passive samplers after experimentation showing the high dust loading
