# Supplementary material for: Passive dust collectors for assessing airborne microbial material
Source: Microbiome. 2015 Oct 5;3:46. doi: 10.1186/s40168-015-0112-7 (PMC4593205; doi:10.1186/s40168-015-0112-7)
Supplement: Additional file 3: — Fungal community composition for the study in USA homes. Description of the experimental methods and results for high-throughput sequencing of the fungi detected in different passive samplers. [file 40168_2015_112_MOESM3_ESM.docx]

Additional file 3: Fungal community composition for the USA homes samples

Fungal community composition was available only for the study of USA homes. Composition analysis followed previously published methods [1]. Briefly, the ITS1 region of the fungal ribosome was targeted using primers adapted for the Illumina MiSeq platform [2]. Bioinformatic analysis relied on a combination of scripts including Trimmomatic [3], USEARCH [4], and QIIME [5].

Figure S1 shows the visualization of fungal community composition across the three homes, and the bacterial composition, copied from the main text, is paired for comparison. Distances in community composition are based on the Bray-Curtis index. Fungal community composition is statistically significantly structured by sampling locality (adonis, p<0.001, R^2^=0.51) but not the height of the sampler or the sampler type (adonis, p>0.05).


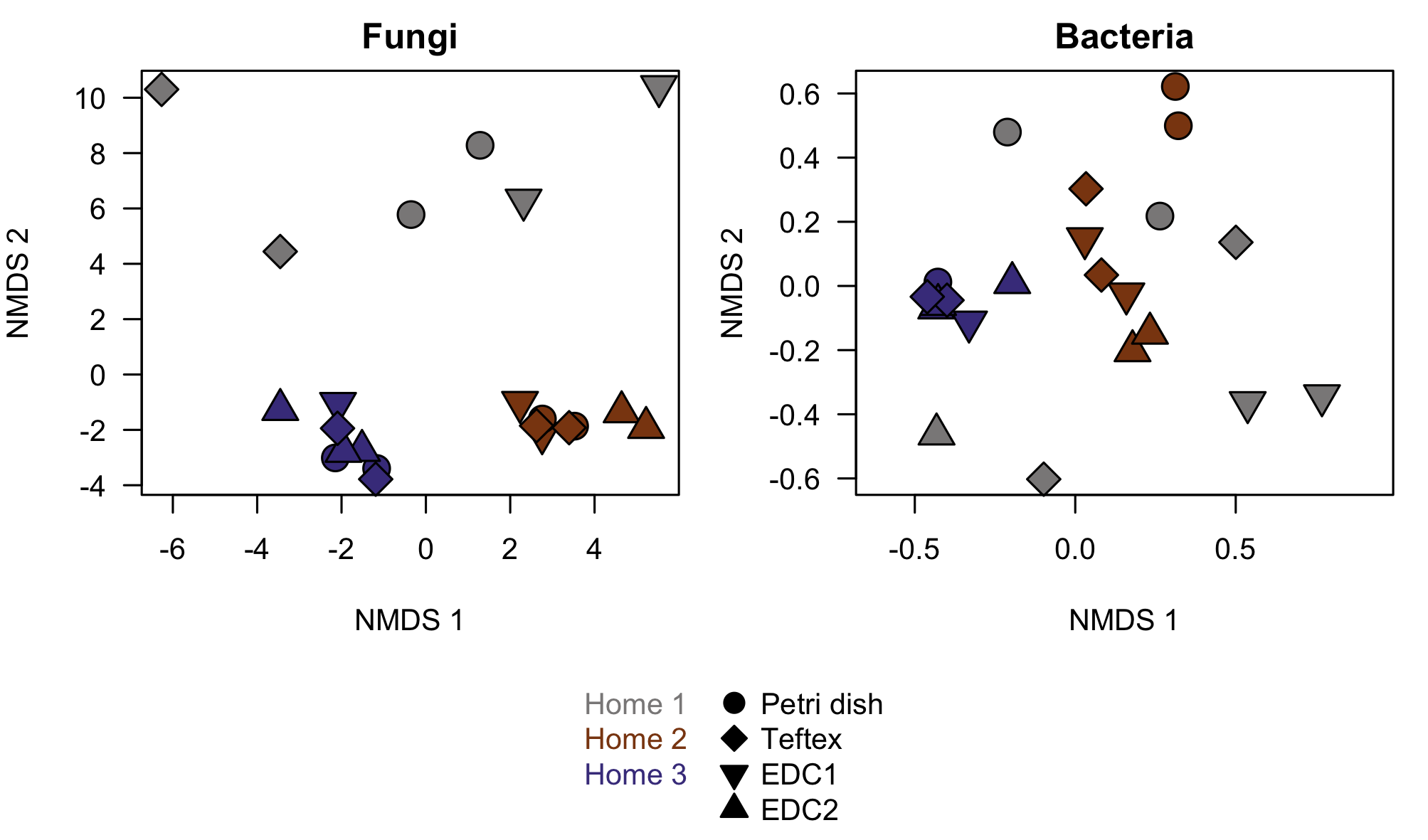


Fig S1. Composition of fungi (left) and bacteria (right) in the USA home. Distances are based on Bray-Curtis community distance for both taxa to facilitate direct comparison. Different houses appear as different colors and different sampling types as shapes. The greater variation in the composition of Home 1 relative to others is likely due to the low biomass in that house, which had low occupancy.

1. Adams RI, Miletto M, Taylor JW, Bruns TD. Dispersal in microbes: Fungi in indoor air are dominated by outdoor air and show dispersal limitation at short distances. ISME J. 2013;7:1262-73. doi:10.1038/ismej.2013.28.

2. Smith DP, Peay KG. Sequence depth, not PCR replication, improves ecological inference from next generation DNA sequencing. PLoS ONE. 2014;9(2):e90234. doi:10.1371/journal.pone.0090234.

3. Bolger AM, Lohse M, Usadel B. Trimmomatic: a flexible trimmer for Illumina sequence data. Bioinformatics. 2014. doi:10.1093/bioinformatics/btu170.

4. Edgar RC. UPARSE: highly accurate OTU sequences from microbial amplicon reads. Nat Methods. 2013;10:996–8. doi:10.1038/nmeth.2604.

5. Caporaso JG, Kuczynski J, Stombaugh J, Bittinger K, Bushman FD, Costello EK et al. QIIME allows analysis of high-throughput community sequencing data. Nat Methods. 2010;7(5):335-6. doi:10.1038/Nmeth.F.303.
