# Supplementary material for: Passive dust collectors for assessing airborne microbial material
Source: Microbiome. 2015 Oct 5;3:46. doi: 10.1186/s40168-015-0112-7 (PMC4593205; doi:10.1186/s40168-015-0112-7)
Supplement: Additional file 4: — Further quantitative characterization of fungi and bacteria with qPCR in Finnish indoor spaces. Details on the complete qPCR measurements taken. Table S1: microbial quantity (cell equivalents/100 cm2 material) as determined through qPCR. Table S2: qPCR geometric mean levels (min-max) of microbial determinations from settled dust utilizing different passive sampling devices in eight homes (duplicate samples). Table S3: intraclass correlations coefficients (ICC) of naturally log-transformed qPCR data for duplicate determinations from eight sample pairs. Table S4: mean coefficient of variations for individual qPCR determination for different sampler types (duplicate samplers of each type in eight homes) and for all qPCR determinations combined. [file 40168_2015_112_MOESM4_ESM.doc]

**Additional file 4: Further quantitative characterisation of fungi and bacteria with qPCR in Finnish indoor spaces**

While estimates of total fungi and gram-positive bacteria appear in the main text, here we report additional estimates from assays of the Penicillium/Aspergillus/Paecilomyces variotii group as well as gram-negative bacteria.

Table S1: Microbial quantity (cell equivalents/100cm^2^ material) as determined through qPCR. Reported are the means, standard deviation (St.Dev), and coefficient of variations (CV) of duplicate (side-by-side) samplers per 100cm^2^ of sampler area. PenAsp = fungal Penicillium/Aspergillus/Paecilomyces variotii; Grampos = Gram positive bacteria; Gramneg = Gram negative bacteria; Unifung = universal fungi.

|  |  | **PetriDish** | | | **Teftex** | | | **EDC** | | |
| --- | --- | --- | --- | --- | --- | --- | --- | --- | --- | --- |
|  |  | Mean | St.Dev | CV | Mean | St.Dev | CV | Mean | St.Dev | CV |
|  |  |  |  |  |  |  |  |  |  |  |
| **Home 1** | PenAsp | 2210 | 1072 | 49% | 737 | 124 | 17% | 1075 | 286 | 27% |
|  | Grampos | 68503 | 31460 | 46% | 11021 | 3110 | 28% | 28689 | 9864 | 34% |
|  | Gramneg | 13944 | 9736 | 70% | 3377 | 668 | 20% | 6579 | 3695 | 56% |
|  | Unifung | 2297 | 419 | 18% | 1235 | 708 | 57% | 1666 | 403 | 24% |
|  |  |  |  |  |  |  |  |  |  |  |
| **Home 2** | PenAsp | 5014 | 74 | 1% | 1796 | 125 | 7% | 3156 | 696 | 22% |
|  | Grampos | 78279 | 7915 | 10% | 25018 | 7706 | 31% | 58885 | 6673 | 11% |
|  | Gramneg | 29564 | 4378 | 15% | 8223 | 847 | 10% | 18734 | 108 | 1% |
|  | Unifung | 2806 | 222 | 8% | 1251 | 295 | 24% | 2125 | 199 | 9% |
|  |  |  |  |  |  |  |  |  |  |  |
| **Home 3** | PenAsp | 17710 | 1563 | 9% | 8862 | 1840 | 21% | 18099 | 643 | 4% |
|  | Grampos | 48072 | 4873 | 10% | 61527 | 7193 | 12% | 51998 | 671 | 1% |
|  | Gramneg | 52773 | 5938 | 11% | 47807 | 4826 | 10% | 58406 | 1195 | 2% |
|  | Unifung | 6775 | 1597 | 24% | 5809 | 164 | 3% | 10719 | 1592 | 15% |
|  |  |  |  |  |  |  |  |  |  |  |
| **Home 4** | PenAsp | 16178 | 3545 | 22% | 5622 | 601 | 11% | 9816 | 2371 | 24% |
|  | Grampos | 85435 | 3892 | 5% | 17187 | 3357 | 20% | 71780 | 12322 | 17% |
|  | Gramneg | 31649 | 157 | 0% | 11582 | 3086 | 27% | 34050 | 10531 | 31% |
|  | Unifung | 5913 | 760 | 13% | 2376 | 363 | 15% | 5315 | 497 | 9% |
|  |  |  |  |  |  |  |  |  |  |  |
| **Home 5** | PenAsp | 6193 | 1593 | 26% | 4712 | 1263 | 27% | 4935 | 23 | 0% |
|  | Grampos | 78516 | 21976 | 28% | 17002 | 3169 | 19% | 39966 | 15787 | 39% |
|  | Gramneg | 65554 | 5762 | 9% | 16589 | 2913 | 18% | 31065 | 6451 | 21% |
|  | Unifung | 9733 | 723 | 7% | 2170 | 117 | 5% | 5768 | 1360 | 24% |
|  |  |  |  |  |  |  |  |  |  |  |
| **Office 1** | PenAsp | 5875 | 2115 | 36% | 748 | 892 | 119% | 6164 | 1585 | 26% |
|  | Grampos | 15235 | 4257 | 28% | 1840 | 2496 | 136% | 4911 | 1109 | 23% |
|  | Gramneg | 33026 | 13494 | 41% | 4439 | 1194 | 27% | 7179 | 4556 | 63% |
|  | Unifung | 1513 | 536 | 35% | 179 | 9 | 5% | 1117 | 18 | 2% |
|  |  |  |  |  |  |  |  |  |  |  |
| **Office 2** | PenAsp | 699 | 173 | 25% | 445 | 603 | 136% | 715 | 443 | 62% |
|  | Grampos | 18569 | 16534 | 89% | 2834 | 84 | 3% | 11259 | 1529 | 14% |
|  | Gramneg | 8359 | 3629 | 43% | 4528 | 4583 | 101% | 6728 | 1039 | 15% |
|  | Unifung | 741 | 459 | 62% | 329 | 33 | 10% | 669 | 85 | 13% |
|  |  |  |  |  |  |  |  |  |  |  |
| **Labspace** | PenAsp | 365 | 141 | 39% | 136 | 168 | 123% | 167 | 81 | 48% |
|  | Grampos | 6243 | 1117 | 18% | 2186 | 1827 | 84% | 2048 | 296 | 14% |
|  | Gramneg | 2216 | 1365 | 62% | 2070 | 2270 | 110% | 62 | 20 | 33% |
|  | Unifung | 392 | 47 | 12% | 179 | 91 | 51% | 156 | 85 | 55% |

Three qPCR determinations (2 Penicillium/Aspergillus qPCR, 1 Grampositive qPCR) were below the limit of quantification; all of those were from samples collected with Teftex material. Table S2 reports the geometric mean levels of microbial biomass by the different passive samplers.

Table S2. qPCR geometric mean levels (min-max) of microbial determinations from settled dust utilizing different passive sampling devices in 8 homes (duplicate samples).

| qPCR assay | Petri dish  (N=16) | Teftex  (N=16) | EDC3  (N=16) |
| --- | --- | --- | --- |
| Total fungi | 2401  (392-9733) | 914  (179-5809) | 1847  (156-10719) |
| Penicillium/Aspergillus spp. | 3620  (365-17710) | 1392  (136-8862) | 2616  (167-18099) |
| Gram positive bacteria | 36539  (6243-85435) | 9312  (1840-61527) | 20273  (2048-71780) |
| Gram negative bacteria | 20190  (2216-65554) | 7555  (2070-47807) | 8308  (62-58406) |

Table S3. Intraclass correlations coefficients (ICC) of naturally log transformed qPCR data for duplicate determinations from 8 sample pairs.

| **qPCR assay** | **N**  **duplicates** | **Petri dish** | **Teftex** | **EDC3** |
| --- | --- | --- | --- | --- |
| **Total fungi** | 8 | 93% | 95% | 97% |
| **Penicillium/Aspergillus spp.** | 8 | 95% | 57% | 95% |
| **Gram positive bacteria** | 8 | 83% | 64% | 97% |
| **Gram negative bacteria** | 8 | 87% | 67% | 97% |
| **All qPCRs** | **32** | **95%** | **75%** | **97%** |

Table S4. Mean coefficient of variations (CV %) for individual qPCR determination for different sampler types (duplicate samplers of each type in eight homes), and for all qPCR determinations combined.

| **qPCR assay** | **N**  **duplicates** | **Petri dish** | **Teftex** | **EDC3** |
| --- | --- | --- | --- | --- |
| **Total fungi** | 8 | 22% | 21% | 19% |
| **Penicillium/Aspergillus spp.** | 8 | 26% | 57% | 27% |
| **Gram positive bacteria** | 8 | 29% | 41% | 19% |
| **Gram negative bacteria** | 8 | 31% | 40% | 28% |
| **All qPCRs** | **32** | **27%** | **40%** | **23%** |

Strong correlation of microbial determinations was observed between sampler types: petridish vs. EDC3 - 0.92; petridish vs. Teftex - 0.92; Teftex vs EDC3 - 0.87(Pearsons correlations, naturally log transformed data, mean values per duplicate samplers).
