# Supplementary figures and images for: Passive dust collectors for assessing airborne microbial material
Source: Microbiome. 2015 Oct 5;3:46. doi: 10.1186/s40168-015-0112-7 (PMC4593205; doi:10.1186/s40168-015-0112-7)

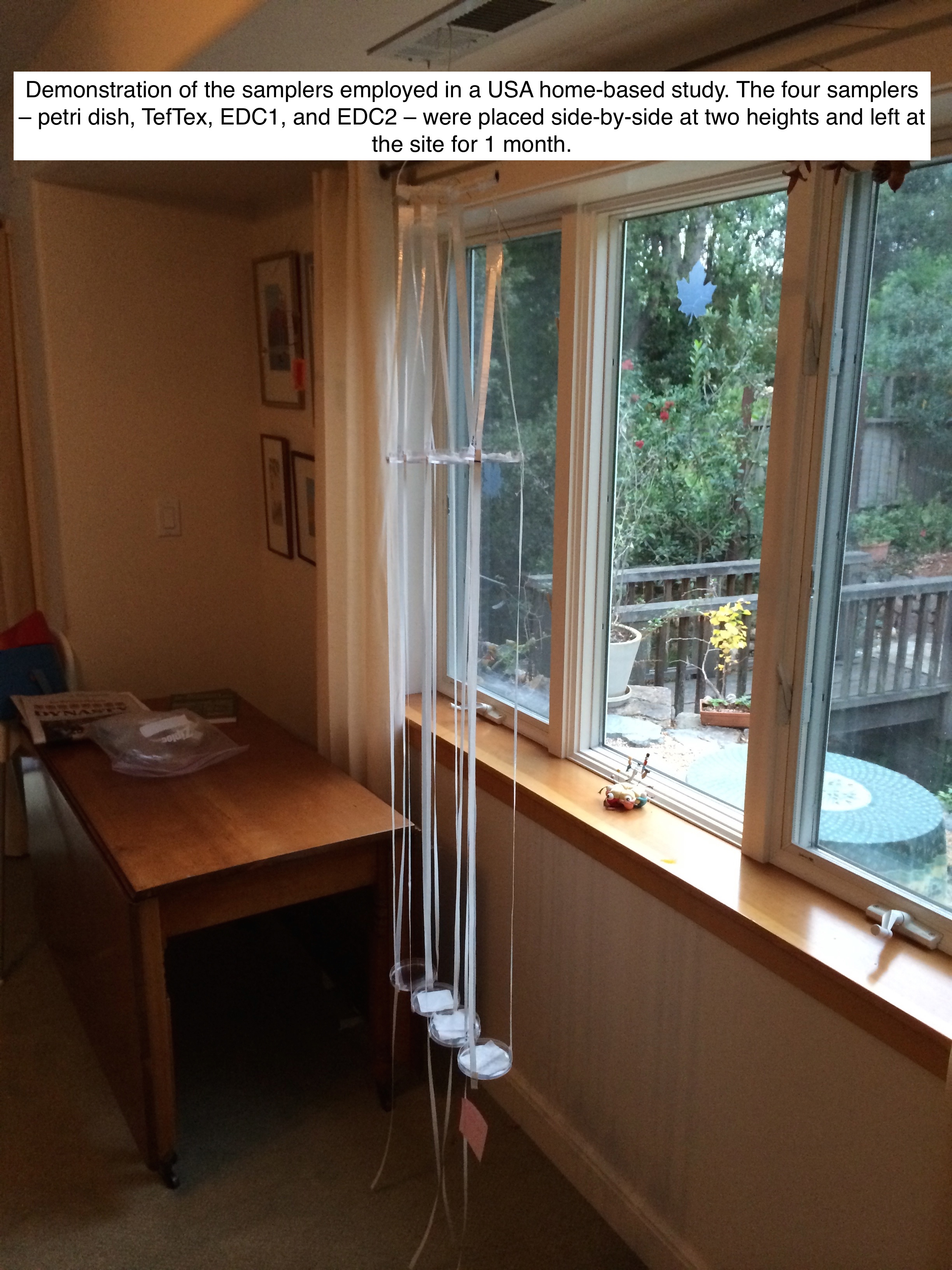

Supplement: Additional file 5: — Picture of the samplers deployed in USA house 2. Demonstration of the samplers employed in a USA home-based study. [file 40168_2015_112_MOESM5_ESM.jpg]
